# Novel Expression Patterns of Metabotropic Glutamate Receptor 6 in the Zebrafish Nervous System

**DOI:** 10.1371/journal.pone.0035256

**Published:** 2012-04-16

**Authors:** Ying-Yu Huang, Marion F. Haug, Matthias Gesemann, Stephan C. F. Neuhauss

**Affiliations:** Institute of Molecular Life Sciences, Neuroscience Center Zürich and Center for Integrative Human Physiology, University of Zürich, Zürich, Switzerland; Institut National de la Santé et de la Recherche Médicale, France

## Abstract

The metabotropic glutamate receptor 6 (mGluR6 or GRM6) belongs to the class III of the metabotropic glutamate receptor family. It is the only known mGluR that mediates direct synaptic transmission in the nervous system and is thought to mediate the ON-response in the ON-pathway of the vertebrate retina. Phylogenetic and gene structure analysis indicated that the zebrafish genome harbours two *mglur6* paralogs, *mglur6a* and *mglur6b*. Besides expression in the inner nuclear layer and distinct regions in the brain, both *mglur6* paralogs are expressed in ganglion cells of the retina, an expression pattern which can also be observed in the downstream effector molecules gnaoa and gnaob. This unexpected expression pattern is consistent with immunohistological labeling using a peptide antibody specific for the mGluR6b paralog. These expression patterns contradict the existing view that mGluR6 is solely located on ON-bipolar cells where it functions in signal transmission. Consistent with expression in ON-bipolar cells, we report a decreased b-wave amplitude in the electroretinogram after morpholino-based downregulation of mGluR6b, showing a function in the ON response. Our data suggest more widespread functions of mGluR6 mediated signaling in the central nervous system, possibly including sign reversing synapses in the inner retina.

## Introduction

Like in other parts of the central nervous system, glutamate is known to be the major excitatory neurotransmitter in the vertebrate retina. The main types of neurons in the retina that use glutamate as a neurotransmitter are photoreceptors (rods and cones), bipolar cells (ON and OFF types), and ganglion cells. These cells form the vertical pathway to convey visual information from the retina to the brain, specifically from photoreceptors via bipolar cells to ganglion cells. Both rod and cone photoreceptors tonically release glutamate in darkness. Upon light exposure they hyperpolarize due to the closure of cation channels leading to a light dependent reduction of glutamate release.

Already at the first visual synapse this signal is parceled into two parallel streams of information: the ON-pathway that is activated by an increase in light (decrease in glutamate) and the OFF-pathway that in turn is activated by a decrease in light (increase in glutamate). Consequently bipolar cells of these two pathways express different glutamate receptors.

The OFF-response is mediated by non-NMDA (AMPA/kainate) ionotropic glutamate receptors expressed on OFF-bipolar cells [1]. This sign-conserving synaptic transmission functions similar to many other excitatory synapses of the central nervous system. In contrast, ON-bipolar cells mediate a sign-reversed signal by being hyperpolarized by glutamate. The glutamate analog 2-amino-4-phosphonobutyric acid (L-AP4 or APB), an agonist to group III metabotropic glutamate receptors, was first discovered as an agonist for ON-bipolar cells [2], [3]. Intracellular electrophysiological recordings demonstrated that APB selectively blocks the light response of ON-bipolar cells in the mudpuppy [3] and rabbit [4] retina, mimicking glutamate release in darkness. Subsequent studies identified the metabotropic glutamate receptor 6 (mGluR6) as the receptor mediating the ON-response. In mammals this receptor is exclusively expressed on the dendrites of ON-bipolar cells [5], where it interacts with the effector G protein Goα [6], [7]. Consistently, genetic inactivation of mGluR6 in mice blocks the ON-response [8]. In humans, mutations in the mGluR6 gene (GRM6) have been linked to congenital stationary night blindness, characterized in the electroretinogram by the absence of an ON response [9], [10].

Upon glutamate binding, these APB-sensitive receptors close cation channels, causing the cells to hyperpolarize [11]. Recently, at least one of these channels has been identified as the transient receptor potential channel M1 (TrpM1) [12], [13], [14].

In the present study we report the identification of two *mglur6* paralogs in the zebrafish genome. The true identity of the two *mglur6* orthologs were confirmed by sequence alignment, genomic structure, and phylogenetic analysis. Subsequent expression studies identified *mglur6* and *gnao* (the gene coding for Goα) expression in the inner nuclear layer, and more surprisingly, strong expression in ganglion cells in both, larval and adult retinas. We confirmed this unexpected finding by immunohistochemistry using a paralog specific peptide antibody against mGluR6b. These data suggest the exciting and unexpected possibility that sign reversing synapses are also present in the inner retina, in addition to the well described role in the outer retina. Indeed, morpholino antisense based knockdown of mGluR6b leads to a reduction in the ON response recorded in the electroretinogram, consistent with findings in other organisms. Additionally, we also found expression in confined brain regions, suggesting a novel, so far unappreciated, role of mGluR6 signaling in retinal ganglion cells and other parts of the brain.

## Results and Discussion

### Molecular cloning and identification of zebrafish metabotropic glutamate receptor type 6

**Figure 1:**
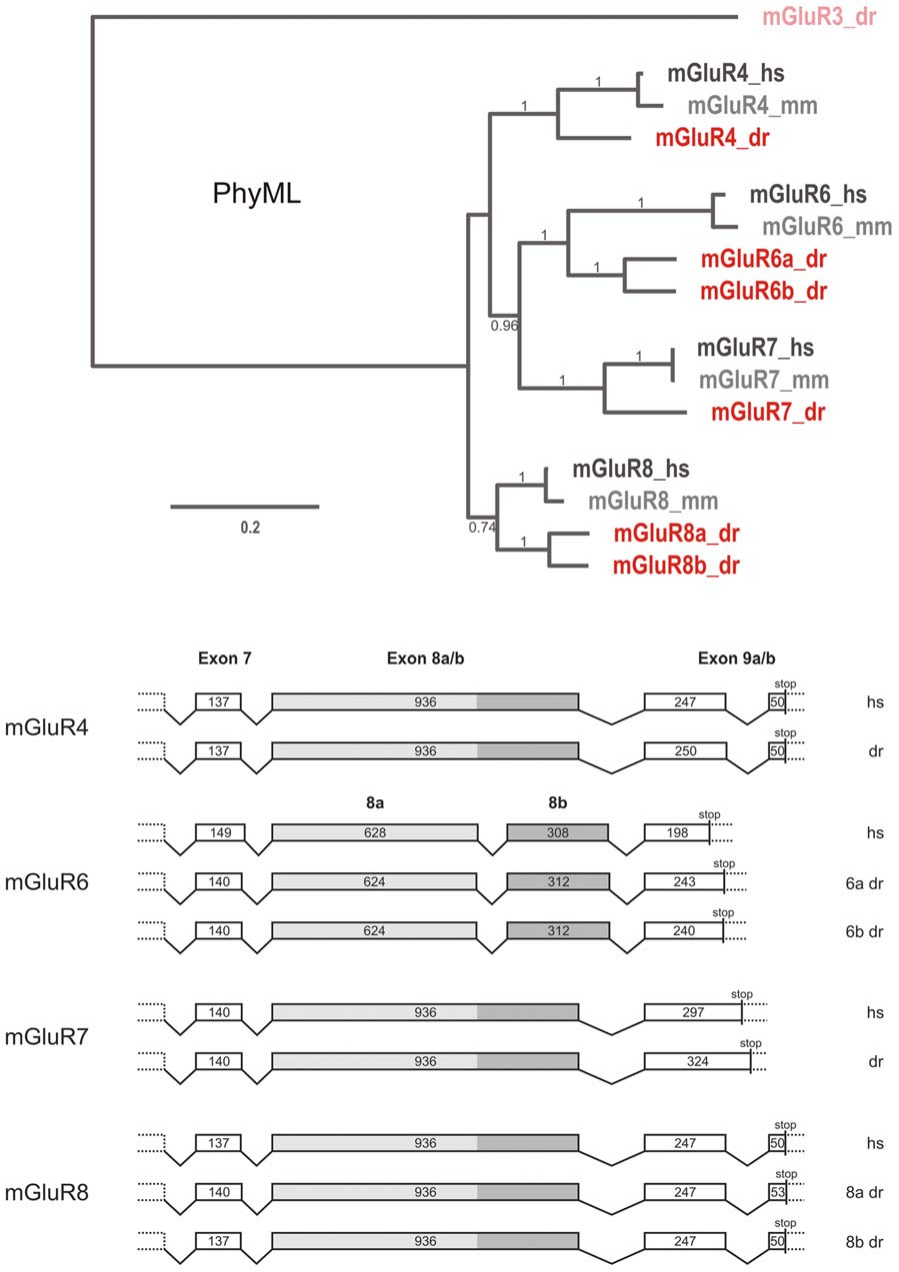
Phylogenetic Reconstruction of mGluR class III genes. *mGluR* sequences of class III metapotropic glutamate receptors of the following species were used in phylogenetic reconstructions (hs = *Homo sapiens*; mm = *Mus musculus* and dr = *Danio rerio*). Sequences were aligned using MUSCLE. A conserved stretch of 746 amino acids determined by the program Gblocks was used for phylogenetic reconstruction. The phylogenetic tree was build using the maximum likelihood method with the WAG amino acid replacement matrix. LRT values above 0.5 are shown. While zebrafish *mglurs* are shown in dark red, mouse *mGluRs* are given in light gray and human mGluRs in dark gray. The genomic organization of mGluR6 differs from other class III *mGluRs*. Analysis of the last exons within the *class III mGluRs* reveals an exon split and a reduced coding sequence length in mGluR6. While the first 7 exons within the class III mGluR subfamily are highly conserved in length, the 936 bp exon 8 found in mGluR4, mGluR7 and mGluR8 is split in mGluR6 (628 bp+308 bp). Moreover the sequence length following the split exons is significantly reduced. Human and zebrafish sequence identity is indicated.

The high sequence homology between class III metabotropic glutamate receptors (*mglur4*, *mglur6*, *mglur7* and *mglur8*) makes it a challenging task to unequivocally identify the true zebrafish *mglur6* ortholog. We identified and manually annotated six unique sequences in the zebrafish genome that clearly belong to class III metabotropic glutamate receptors. On the protein level the identity between these sequences ranged from 71 to 89% (Table 1). In order to assign these sequences to the various members of the subgroup, we performed a phylogenetic analysis using these sequences with the published sequences of the mouse and human group III orthologs (Figure 1A). In this way we identified one zebrafish *mglur4* and *mglur7* ortholog each, while *mglur6* and *mglur8* have two paralogous genes per mammalian ortholog. These paralogs reside on different chromosomes and are likely remnant of the teleost specific genome duplication event [26], [27]. We gained further support for the correct assignment of *mglur6* sequences by comparing the genomic structure of the class III genes. Interestingly, with the exception of *mglur6*, the length of exon 8 is 936 bp in all class III *mglur* genes. However, in *mglur6* this exon is split into two exons. The length of these two split exons add up to yield the conserved length of exon 8 (Figure 1B). Hence this exonal split likely predates the origin of vertebrates about 500 million years ago.

**Table 1:** Pairwise alignment of protein sequences of human (hs), and zebrafish (dr) class III mGluRs.

mGluR4_dr	mGluR6_hs	mGluR6a_dr	mGluR6b_dr	mGluR7_hs	mGluR7_dr	mGluR8_hs	mGluR8a_dr	mGluR8b_dr	
**81** (90)	**71** (83)	**72** (85)	**73** (85)	**71** (85)	**70** (85)	**77** (87)	**74** (85)	**75** (85)	**mGluR4_hs**
	**69** (83)	**72** (85)	**72** (84)	**70** (83)	**71** (84)	**76** (85)	**74** (85)	**74** (85)	**mGluR4_dr**
		**75** (86)	**76** (87)	**68** (83)	**69** (83)	**72** (83)	**71** (83)	**71** (83)	**mGluR6_hs**
			**87** (95)	**73** (88)	**73** (87)	**75** (87)	**74** (86)	**73** (87)	**mGluR6a_dr**
				**73** (87)	**73** (86)	**74** (86)	**73** (86)	**73** (86)	**mGluR6b_dr**
					**83 (91)**	**76** (87)	**73** (86)	**73** (86)	**mGluR7_hs**
						**74** (85)	**73** (86)	**71** (85)	**mGluR7_dr**
							**85** (92)	**83** (92)	**mGluR8_hs**
								**89** (94)	**mGluR8a_dr**

The percentage of identical amino acids between sequences is given in bold numbers, whereas the percentage of conserved amino acids is given in parenthesis. Conservation between orthologs is emboldened.

### Embryonic and adult gene expression of zebrafish *mglur6a* and *mglur6b*

**Figure 2:**
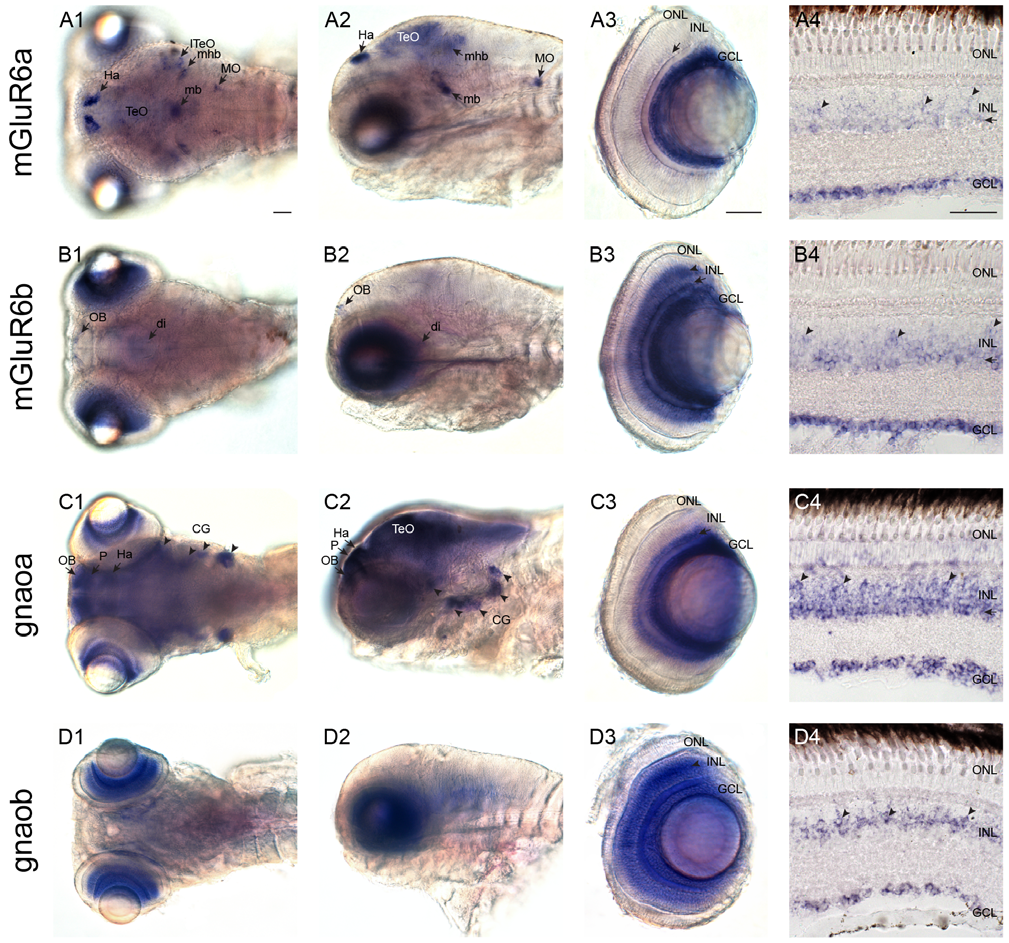
RNA expression of zebrafish *mglur6 and gnao* paralogs. RNA expression of *mglur6a* (A1–A4), *mglur6b* (B1–B4), *gnaoa* (C1–C4) and *gnaob* (D1–D4) in dorsal (1) and lateral (2) views and in isolated eyes (3) of a 5 dpf zebrafish, as well as in adult retinal cross-sections (4). **A1, A2:** Expression of *mglur6a* is visible in the habenula (Ha), the medial (TeO) and lateral (lTeO) tectum opticum, the midbrain (mb), a part of the mid-hindbrain boundary (mhb) and a bilateral nucleus of the medulla oblongata (MO). **A3:** In an eye separated from a whole mount stained larva *mglur6a* is expressed in the proximal inner nuclear layer (INL, arrow) and the ganglion cell layer (GCL). **A4:** Additional to the cellular expression in the proximal INL (arrows) and the GCL, *mglur6a* labels cells in the medial INL (arrowheads) in adult. **B1, B2:** *mglur6b* reveals a staining in the olfactory bulb (OB) and a weak labeling of a part of the diencephalon (di). **B3:** The isolated eye shows, *mglur6b* expression in the medial INL (arrowhead), the proximal INL (arrow) and the GCL. **B4:** The adult retinal cross section shows the same localization for *mglur6b* as the larval fish, however, the staining in the medial INL is restricted to a subset of cells (arrowheads). **C1, C2:** *gnaoa* is expressed in the olfactory bulb (OB), the pallium (P), the habenula (Ha), the tectum opticum (TeO) and in all cranial ganglia (CG, arrowheads). **C3:** The retina reveals *gnaoa* labeling in the proximal INL (arrow) and the GCL. **C4:** Similar to the larval retina, *gnaoa* labels the proximal INL (arrow) and the GCL in the adult retina. Additionally, *gnaoa* stains weakly a subset of cells in the medial INL (arrowheads). **D1, D2:** *gnaob* shows no expression in the brain. **D3:** The expression of *gnaob* in the larval fish eye is restricted to the medial INL (arrow) and the GCL. **D4:** Similar to the larval eye, in adult retinal cross-sections *gnaob* is located in cells of the medial INL and the GCL. All scale bars = 40 µm. Scale bar in A1 applies to all whole mount images, scale bar in A3 applies to A3, B3, C3 and D3, scale bar in A4 applies to A4, B4, C4 and D4.

Surprisingly, both *mglur6* paralogs revealed a predominant expression in the ganglion cell layer (GCL) in larval (Figure 2A3, B3) as well as in adult fish (Figure 2A4, B4). Moreover, both larval and adult retinas, expressed small amounts of *mglur6a* and -*6b* RNA in the proximal inner nuclear layer (INL) close to the inner plexiform layer (IPL) (arrow, Figure 2A3, A4, B3, B4). An additional expression in the medial INL is visible in retinas stained with the *mglur6b* riboprobe (arrowhead, Figure 2B3, B4), however, *mglur6a* expression in this layer seems to be restricted to adult fish (arrowhead, Figure 2A4).

**Figure 3:**
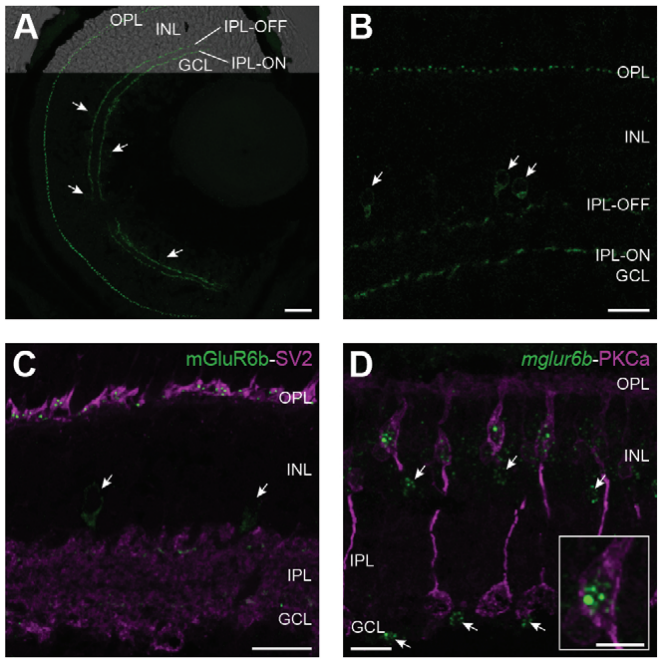
Subcellular localization of mGluR6b in the zebrafish retina. Z-projections of confocal image stacks of immunohistochemically labeled larval and adult retinal cross-sections. **A:** A larval retina at 5 dpf stained with the mGluR6b antibody shows labeling in the outer plexiform layer (OPL) and in an ON- and an OFF-layer of the inner plexiform layer (IPL). In addition, single cells adjacent to the IPL in the INL and the GCL (arrowheads) express mGluR6b as well. Scale bar = 20 µm. **B:** In the adult zebrafish retina, mGluR6b stains similar structures as in the larval retina with the exception that we could only detect labeled cells in the inner part of the INL (arrowhead) but not in the GCL. Scale bar = 15 µm. **C:** A doublelabeling of mGluR6b (green) and SV2 (magenta) in an adult retinal cross-section reveals the postsynaptic localization of mGluR6b in the OPL. Again, an mGluR6b expression in single cells of the proximal IPL is detected (arrows). Scale bar = 15 µm. **D:** Fluorescent *in situ* hybridization of *mglur6b* (green) combined with an immunohistochemical labeling of ON-bipolar cells by PKCalpha (magenta) confirms the localization of mGluR6b in ON-bipolar cells of the INL. Scale bar = 15 µm. Arrows point to cells of the proximal INL and the GCL expressing *mglur6b*. The insert shows a close up of an ON-bipolar cell body and its fluorescent *mglur6b* signal in the cytosol. Scale bar = 5 µm.

In order to additionally confirm these results and localize the encoded protein, we raised paralog specific polyclonal antibodies against mGluR6b. We detected mGluR6b immunoreactivity in the outer plexiform layer and in both ON-and OFF-layer of the IPL. Additionally, single cells adjacent to the IPL in both INL and GCL were labeled with the mGluR6b antibody (Figure 3A). In the adult retina we found a similar expression with the exception that we did not see labeled cells in the GCL (Figure 3B). In order to confirm the postsynaptic localization of mGluR6b protein in the OPL, we co-stained adult retinal sections with antibodies against mGluR6b and the presynaptic marker SV2 (Figure 3C). Finally, we performed a fluorescent *in situ* hybridization on sections with PKCalpha immunolabeled ON-bipolar cells, to additionally confirm the expression of *mglur6b* in ON-bipolar cells (Figure 3D).

These results contrast with most studies in other vertebrates that find mGluR6 exclusively expressed in ON-bipolar cells [5], [28], [29]. However, few studies report *mglur6* expression in juvenile and regenerating rat retinal ganglion cells [30]. Recently, the expression of mGluR6 mRNA in some human ganglion cells has been reported [31].

There is good physiological evidence that the ON-response of the rod pathway [32], [33] and to a lesser extent also the cone ON-pathway [33], [34] is mediated at least partially by mGluR6 signaling in the zebrafish.

In order to demonstrate a role of mGluR6b in mediating the cone ON-response, we downregulated mGluR6b by morpholino antisense injections. We found a concentration dependent reduction of the ERG b-wave, confirming a role of mGluR6b in mediating the cone ON-response, since at this stage (5 dpf) most if not all of the visual response is cone mediated. We verified the efficiency of our knockdown by showing a concomitant loss of immunohistochemical staining (Figure S1). Since we recorded a small b-wave at the highest non-toxic concentrations where all mGluR6b protein staining was abolished, we suggest that at least one other glutamate receptor system may be involved in the cone ON response in the zebrafish. The mGluR6a paralog may mediate this remaining ON response, but we deem it more likely that a mGluR6 independent pathway accounts for part of the ON response.

At least in lower vertebrates there is good evidence that the cone ON- response is predominantly mediated by an excitatory amino acid transporter (EAAT) mediated mechanism [33], [35], [36].

The strong expression of both *mglur6* paralogs in the ganglion cell layer during larval and adult stages implies an additional function of mGluR6 signaling cascade in this cell layer. In order to find supportive evidence for this unexpected result, we cloned the downstream effector G protein Goα subunit, which is part of the mGluR6 signaling cascade [6], [7]. We identified the two zebrafish paralogs *gnaoa* and *gnaob* whose expression pattern in the retina is very similar to the *mglur6* paralogs. The retinal location of *gnaoa* in larval and adult zebrafish (Figure 2C3, C4) is similar to the expression of *mglur6a*, whereas *gnaob* is located in the medial INL already in larval fish (Figure 2D3) – comparable to *mglur6b*. Like the *mglur6* paralogs, both *gnao* paralogs were expressed in retinal ganglion cells (Figure 2C3, C4, D3, D4). These results are interesting in the context of nyctalopin. This enigmatic protein is directly involved in the mGluR6 signaling cascade and likely interacts with both mGluR6 and TrpM1 protein [37]. Consistent with the presence of a functional mGluR6 signaling cascade, we found *nyctalopin* expression in larval and adult retinal ganglion cell layer ([38] and Edda Kastenhuber, personal communication). Interestingly a transgenic zebrafish line expressing yellow fluorescent protein under the control of about 1.5 kb upstream of the start codon of the *nyctalopin* gene lacks expression in ganglion cells [39]. Presumably regulatory regions needed for retinal ganglion cell expression are located farther away from the coding sequence.

Equally surprising, we found *mglur6* expression outside of the retina. The larval zebrafish brain shows *mglur6a* labeling in the habenula (Ha), medial and lateral tectum opticum (TeO, lTeO), midbrain (mb), mid-hindbrain boundary (mhb) and in a bilateral nucleus of the medulla oblongata (MO, Figure 2A1, A2). *mglur6b* expression in the larval brain is confined to the olfactory bulb (OB, Figure 2B1, B2) and a part of the diencephalon (di, Figure 2B1, B2). These results point to the exciting possibility of more widespread use of mGluR6 signaling, potentially in sign reversing synapses, not only in the inner retina but also in the central nervous system in general. This hypothesis is substantiated by the expression of Gαo in these brain regions.

In future experiments, it will be important to follow up these findings in the mammalian brain, where so far there are only two reports on the expression of *mglur6* outside of the retina [40], [41].

In conclusion, we have identified two paralogous *mglur6* genes in the zebrafish genome. Apart from expression in the inner nuclear layer of the retina, we found strong expression in retinal ganglion cells and in other parts of the brain, suggesting a more widespread use of the mGluR6 signaling cascade in the central nervous system.

## Materials and Methods

### Fish Maintenance and breeding

Fish were maintained and bred as previously described [15] and kept under a 14 h/10 h light/dark cycle. The wild-type strain used for all studies was WIK. Embryos were raised at 28°C in E3 medium and staged according to development in days post fertilization (dpf). All examinations were performed in accordance with the ARVO Statement for the Use of Animals in Ophthalmic and Vision Research and were approved by the local authorities (Veterinäramt Zürich TV4206).

### Cloning of *mglur6* and *gnao* paralogs

Total mRNA was isolated using the QIAShredder and the RNeasy kit (Qiagen, Hombrechtikon, Switzerland) from about 50 larvae at 5 days post-fertilization (dpf) and reversed transcribed to single strand cDNA using oligo-dT primer (First Strand Kit; Stratagene, La Jolla, CA). Polymerase chain reaction (PCR) was performed with Taq polymerase (Taq Gold; Applied Biosystems, Switzerland) using sequence specific oligonucleotide primers. Amplified DNA pieces were subcloned into TOPO pCRII vectors (Invitrogen, Basel, Switzerland) and sequenced.

### Annotation of *mglur* cDNAs

As gene predictions within GeneBank are produced by automated processes which have been shown to contain numerous errors, mGluR cDNA sequences used in this study were manually annotated. Sequences were identified and annotated using combined information from expressed sequence tags and genome databases (GeneBank, http://www.ncbi.nlm.nih.gov; Ensembl, http://www.ensembl.org/index.html; version 50/51, 2008). Human and mouse sequences were used as initial query (for more details on sequence annotation see [16]).

### Phylogenetic tree analysis

The EditSeq software (Lasergene; DNASTAR, Madison, WI, USA) was used to translate the coding sequences of *mglur* genes into amino acid sequences and the obtained protein sequences were used to generate a combined sequence file in FASTA format. Sequence alignment and phylogenetic analysis was performed on the Phylogeny.fr platform ([17]; http://www.phylogeny.fr/version2_cgi/phylogeny.cgi). Sequences were aligned using muscle (v3.7; [18]) configured for highest accuracy (muscle with default settings). After alignment, ambiguous regions (i.e. containing gaps and/or being poorly aligned) were removed with Gblocks (v0.91b; [19]). The phylogenetic tree was reconstructed by the maximum likelihood method [20], 2003) using the WAG amino acid replacement matrix [21] implemented in the PhyML program (v3.0). The approximate likelihood ratio test (aLRT; [22]) was used to judge branch reliability. Graphical representation and editing of the phylogenetic tree was done using TreeDyn (v198.3) and the obtained svg files were colored using the CorelDraw program.

### Whole mount and transverse cross sections *in situ* hybridization

Sequence-confirmed cDNA clones were used as templates for *in vitro* transcription to produce RNA probes for DIG (digoxigenin labeled) *in situ* hybridization (Roche Diagnostics, Rotkreuz, Switzerland). The used riboprobes spanned the following regions (base pairs counted from the ATG): *mglur6a*: 1345–2425, *mglur6b*: −10–1676, *gnaoa*: 410–1335 or 2221 and *gnaob*: 191–1192 or 193–2059. Sense and antisense RNA probes were synthesized by Sp6 and T7 RNA polymerase (Roche). RNA probes were hydrolyzed to yield fragments of about 300–500 nucleotides in length. Whole-mount *in situ* hybridization was performed on 3 µM PTU (1-phenyl-2-thiourea, Sigma, St. Louis, MO, USA) -treated WIK wild-type larvae. They were collected at 5 dpf, anesthetized on ice, and immediately fixed in 4% paraformaldehyde (PFA) in phosphate buffered saline (PBS, freshly prepared, pH 7.4) over night at 4°C. Whole mount *in situ* hybridization was performed according to Thisse and Thisse [23] with minor modifications: 5-day-old fish were treated with proteinase K (10 µg/ml) for 75 min. From day two on TNT (100 mM Tris HCl pH 7.5, 150 mM NaCl, 0.5% Tween 20) was used for all washing steps instead of PBT (PBS, 0.1% Tween 20). AP-conjugated anti-DIG antibodies (Roche) were diluted 1∶5000 in blocking solution (Roche) in TNT. The staining was stopped with PBT and the larvae were postfixed in 4% PFA in PBS ON at 4°C. Subsequently, larvae were mounted on an adapted glass slide in 100% glycerol (Sigma) and imaged with an Olympus BX61 microscope using a color camera (ColorView IIIu, Soft Imaging System, Olympus). For obtaining cross sections of adult eyes, the eyecups were dissected and, similar to larval fish, fixed over night in 4% PFA at 4°C. After washing twice with PBT for 5 min at RT, the eyes were placed in 30% sucrose and incubated ON at 4°C. Subsequently, the eyecups were embedded in cryomatrix (Tissue Tek O.C.T., Sakura, Zoeterwonde, NL), fast frozen with liquid N_2_ and stored at −80°C until further use. 16 µm thick transverse sections were cut using a Microm microtome (HM 550), collected on glass slides, dried at 37°C for 30 min and stored at −80°C until using them for *in situ* hybridization. *In situ* hybridization was done as described above for larval fish, but proteinase K permeabilization time was reduced to 2.5 min and PBT was used for the washing steps and to dissolve the blocking reagent on day 2. Finally, slides were cover-slipped with Kaiser's glycerol gelatine (Merck KGaA, Darmstadt, Germany) and images were taken with the bright field modus of a light microscope (Olympus BX61). For fluorescent *in situ* hybridization on adult eye sections, an incubation step in 1% H_2_O_2_ in PBT for 20 min prior to the blocking step was included to quench endogenous peroxidase activity. To detect the probe a 1∶400 dilution of anti-DIG-POD antibody (Roche) was used. The next day, the slides were washed several times with PBT and incubated for 1 h in the dark at RT with the tyramide working solution (Invitrogen, TSA Kit *41, Alexa 555). For the subsequent immunostaining, slides were kept in darkness. After three additional washing steps in PBT, slides were treated as previously described [24] but blocking solution was applied for 1 h at RT. For the labeling of ON-bipolar cells we used the mouse anti-PCKalpha (MC5) antibody (Novus Biologicals, NB200-586; 1∶500).

### Generation of antibodies

For immunization, a peptide specific for mGluR6b has been chosen. Rabbits were immunized with the peptide **QKS**SD**KQNGETK**V**EPDR**S**Q** (889–905; amino acids in bold represent sequence identity with zebrafish mGluR6a). The mGluR6b antibody was affinitypurified against the respective peptide by Eurogentec (Eurogentec S.A., Seraing, Belgium).

### Immunohistochemistry

Immunohistological staining was performed as previously described [24], however, blocking solution was only applied for 1 h at RT.

To increase specificity, the rabbit anti-mGluR6b antibody was cross-absorbed against the respective mGluR6a epitope using CNBr-activated sepharose (4B, GE Healthcare, Little Chalfont, UK) and the increased specificity was tested on dot-blots (Figure S2). The final concentration of the cross-absorbed rabbit anti-mGluR6b antibody on the sections was 1∶150. Doublelabelings were performed with mouse anti-synaptic vesicle 2 (SV2; 1∶100; DSHB, Iowa, USA). Pictures were taken with a Leica confocal microscope (Leica SP5, Leica Microsystems GmbH, Wetzlar, Germany). Confocal z-stacks were analyzed by using Imaris (Bitplane, Zürich, Switzerland).

### Targeted gene knockdown (Morpholino)

**Figure 4:**
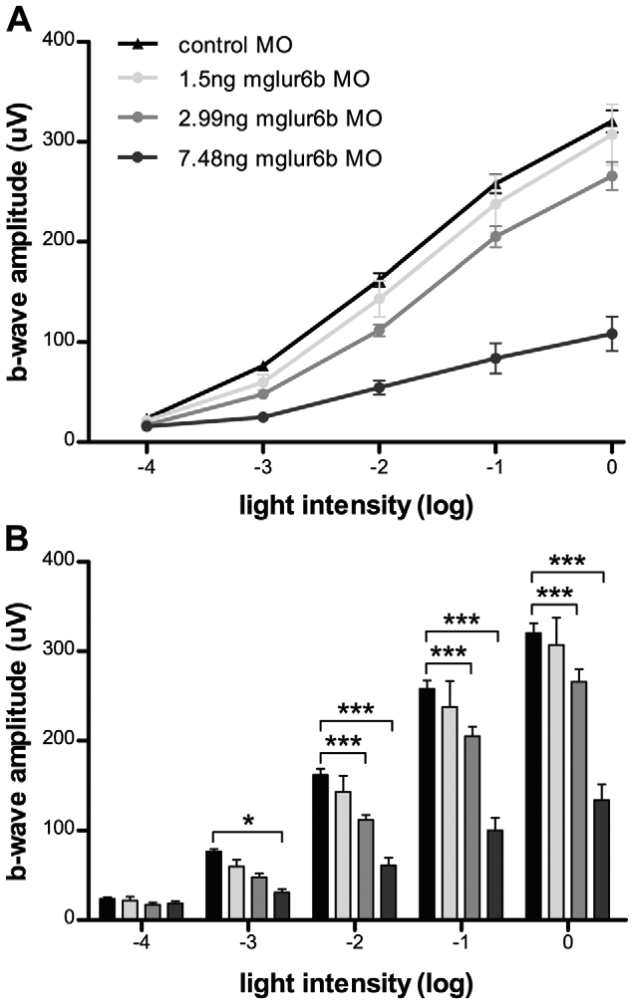
Electroretinogram recordings of *mglur6b*-depleted zebrafish larvae. The downregulation of mGluR6b leads to a dose dependent decrease of the ERG b-wave in 5 day old zebrafish larvae indicating a diminished ON-response. **A:** Plotted b-wave amplitudes at different light intensities. Control Morpholino (MO) injected larvae of different concentrations showed no significant differences in b-wave amplitude, neither among each other nor in comparison to uninjected wild type larvae (data not shown). Therefore, recordings of all control MO larvae (n = 46) were taken together to build one curve. Injection of *mglur6b* MO leads to a dose dependent depletion of the b-wave (1.5 ng *mglur6b* MO: n = 14; 2.99 ng MO: n = 24; 7.48 ng MO: n = 13). All data points represent the means ± SEM. **B:** Significance of the b-wave amplitude reduction was calculated using a 2-way ANOVA (* = p<0.1; ** = p<0.01; *** = p<0.001).

The specific downregulation of *mglur6b* was accomplished by the injection of an antisense morpholino oligonucleotide (MOs; Gene Tools, Philomath, Oregon, USA) designed against the translational start site (5′-TGTGATGTCATAGTTGCTGGCATTC-3′). As a control, a standard MO (5′-CCTCTTACCTCAGTTACAATTTATA-3′) was used. Prior to use, both MOs were dissolved in ddH_2_0 to a stock concentration of 2 mM and stored at −20°C. To obtain a working solution, MOs were diluted in PBS pH 7.4 containing 0.2% phenol red to the desired concentration. Embryos were injected in the one-cell stage with the given concentrations (see Figure 4).

### Electroretinogram

Electroretinogram (ERG) recordings were performed as previously described [25]. Before the recordings, larvae were dark adapted for at least 30 min. Stimuli of 100 ms with an interstimulus interval of 5 s were applied to elicit a response. The light stimulus intensity was 6800 lux or 72 W/m^2^ (for log 0). The measurement series started with the lowest light intensity (log −4).

Statistical analysis was performed in GraphPad Prism 5 (GraphPad Software, La Jolla, CA, USA).

## Supporting Information

Figure S1 **mGluR6b expression in the** ***mglur6b*** **-depleted zebrafish retina at 5 dpf.** Immunohistochemical analysis using the mGluR6b antibody confirms the downregulation of mGluR6b in the 5 dpf zebrafish retina. **A:** mGluR6b expression in a non-injected sibling. **B:** Injection of 2.5 ng *mglur6b* MO leads to an incomplete downregulation of the mGluR6b protein since a faint staining in the plexiform layers is still visible. **C:** 6.7 ng mglur6b antisense-RNA lead to a complete knockdown of the mGluR6b protein in 5 dpf zebrafish retinas. Scale bar (applies for all images A–C) = 20 µm.
(TIF)

Figure S2 **Cross-absorbance of the mGluR6b antibody.** **A:** Immunohistochemistry image of a cross section through a 5 day old larval retina stained with the original mGluR6b antibody (1∶200). **B:** Confocal image of a larval retina at 5 dpf stained with the cross-absorbed mGluR6b antibody (1∶150). For further description see Figure 3. Scale bar in B = 20 µm (applies for A and B). **C:** Dot-blot analysis showing the increased specificity of the cross-absorbed mGluR6b antibody. 1 µg of mGluR6a (6a) and mGluR6b (6b) epitopes were pipetted on nitrocellulose membranes (0.45 µm; Bio-Rad, Reinach, Switzerland) and incubated with the non cross-absorbed and the cross-absorbed antibodies. Following cross-absorbing the epitope of the mGluR6a is not recognized anymore.
(TIF)
