# Supplementary figures and images for: Novel Expression Patterns of Metabotropic Glutamate Receptor 6 in the Zebrafish Nervous System
Source: PLoS One. 2012 Apr 16;7(4):e35256. doi: 10.1371/journal.pone.0035256 (PMC3327648; doi:10.1371/journal.pone.0035256)

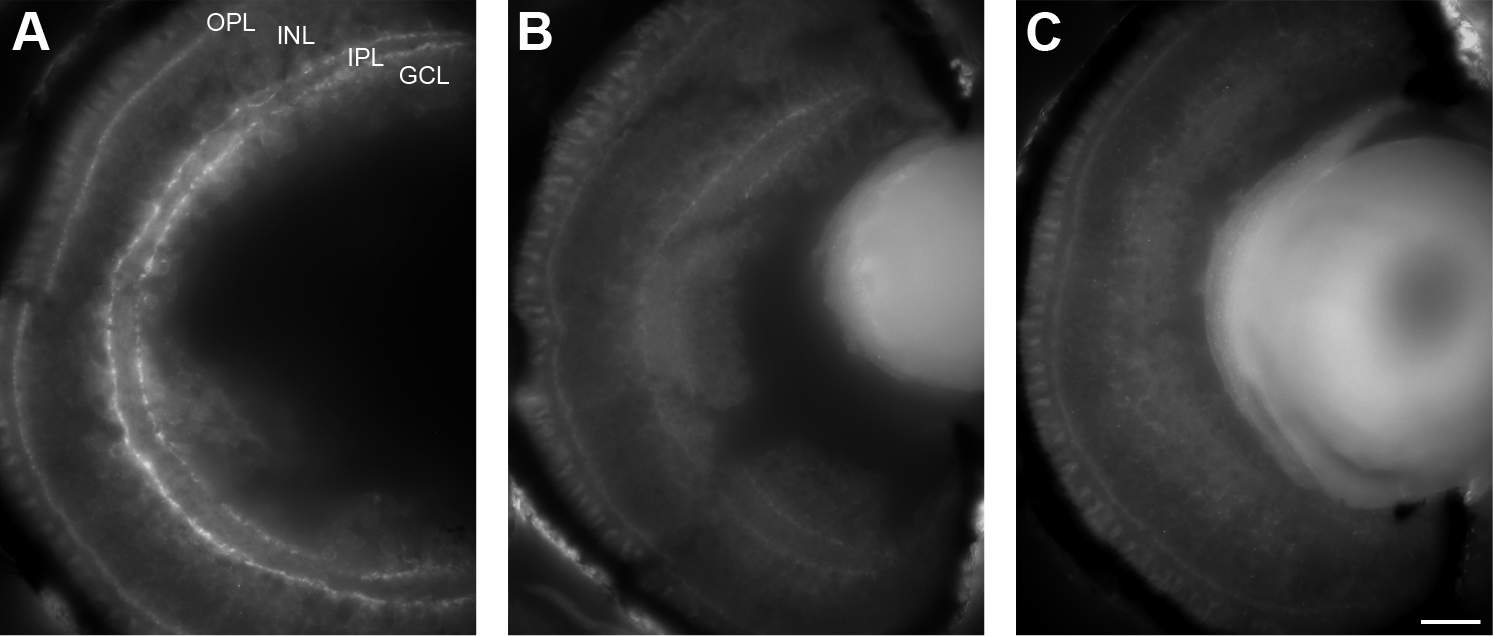

Supplement: Figure S1 — mGluR6b expression in the mglur6b -depleted zebrafish retina at 5 dpf. Immunohistochemical analysis using the mGluR6b antibody confirms the downregulation of mGluR6b in the 5 dpf zebrafish retina. A: mGluR6b expression in a non-injected sibling. B: Injection of 2.5 ng mglur6b MO leads to an incomplete downregulation of the mGluR6b protein since a faint staining in the plexiform layers is still visible. C: 6.7 ng mglur6b antisense-RNA lead to a complete knockdown of the mGluR6b protein in 5 dpf zebrafish retinas. Scale bar (applies for all images A–C) = 20 µm. (TIF) [file pone.0035256.s001.tif]

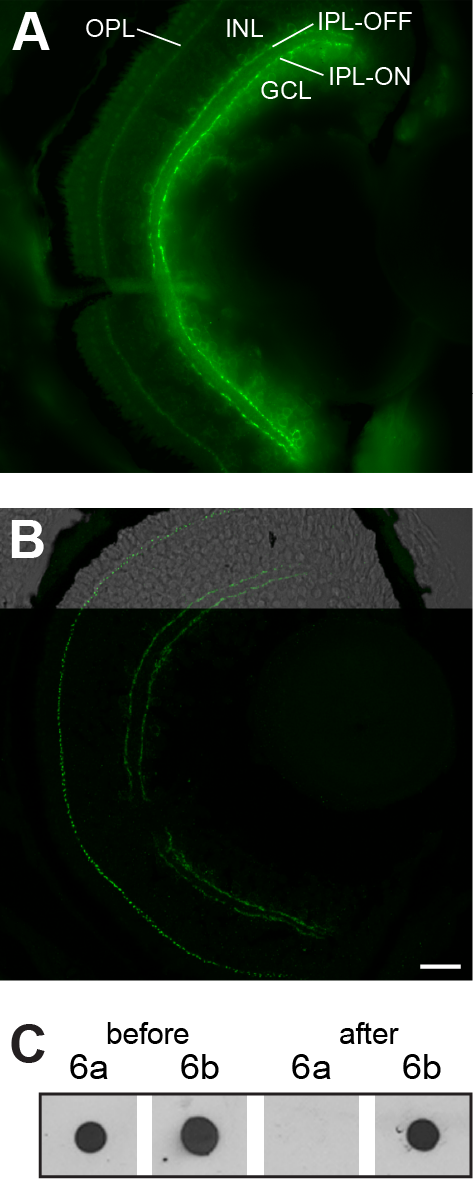

Supplement: Figure S2 — Cross-absorbance of the mGluR6b antibody. A: Immunohistochemistry image of a cross section through a 5 day old larval retina stained with the original mGluR6b antibody (1∶200). B: Confocal image of a larval retina at 5 dpf stained with the cross-absorbed mGluR6b antibody (1∶150). For further description see Figure 3. Scale bar in B = 20 µm (applies for A and B). C: Dot-blot analysis showing the increased specificity of the cross-absorbed mGluR6b antibody. 1 µg of mGluR6a (6a) and mGluR6b (6b) epitopes were pipetted on nitrocellulose membranes (0.45 µm; Bio-Rad, Reinach, Switzerland) and incubated with the non cross-absorbed and the cross-absorbed antibodies. Following cross-absorbing the epitope of the mGluR6a is not recognized anymore. (TIF) [file pone.0035256.s002.tif]
